# Geochemical Distribution and Environmental Risks of Radionuclides in Soils and Sediments Runoff of a Uranium Mining Area in South China

**DOI:** 10.3390/toxics12010095

**Published:** 2024-01-22

**Authors:** Haidong Li, Qiugui Wang, Chunyan Zhang, Weigang Su, Yujun Ma, Qiangqiang Zhong, Enzong Xiao, Fei Xia, Guodong Zheng, Tangfu Xiao

**Affiliations:** 1 State Key Laboratory of Nuclear Resources and Environment, East China University of Technology, Nanchang 330013, China; hdli0828@163.com (H.L.); fxia@ecut.edu.cn (F.X.); 2 Research Institute No. 290, China National Nuclear Corporation, Shaoguan 512029, China; zgd0406@163.com; 3 Key Laboratory for Water Quality and Conservation of the Pearl River Delta, Ministry of Education, School of Environmental Science and Engineering, Guangzhou University, Guangzhou 510006, China; xiaoez@gzhu.edu.cn (E.X.); tfxiao@gzhu.edu.cn (T.X.); 4 Key Laboratory of Tibetan Plateau Land Surface Processes and Ecological Conservation (Ministry of Education), Qinghai Normal University, Xining 810008, China; yujunma@qhnu.edu.cn; 5 Disaster Reduction and Disaster Preparedness Center of Jiangxi, Nanchang 330036, China; 13047903981@163.com; 6 Key Laboratory of Comprehensive and Highly Efficient Utilization of Salt Lake Resources, Chinese Academy of Sciences and Environment of Salt Lakes, Qinghai Institute of Salt Lakes, Chinese Academy of Sciences, Xining 810016, China; 7 Third Institute of Oceanography, Ministry of Natural Resources, Xiamen 361005, China; zhongqiangqiang@tio.org.cn

**Keywords:** natural radionuclides, granite uranium mining areas, radiological hazards, river sediments and soils

## Abstract

Uranium mining activities have contributed to the distribution and uptake of radionuclides, which have increased the active concentrations of natural radionuclides in environmental media, causing elevated human health risks. The present study aims to assess the spatial distribution characteristics of natural radionuclides in the surface soils and river sediments of the typical granite uranium mining area in South China, as well as investigate the geochemical features of natural radionuclides in the soil and sediments to understand their migration processes. The activity concentrations for ^238^U, ^226^Ra, ^232^Th, and ^40^K ranged from 17–3925 Bq/kg, 50–1180 Bq/kg, 29–459 Bq/kg, and 240–1890 Bq/kg, respectively. The open-pit mining areas and tailings pond locations exhibited the highest concentrations of activity for all these radionuclides. This distribution points to an elevated potential health risk due to radiological exposure in these specific areas. Additionally, the values of radium equivalent activity (Ra_eq_) and annual gonadal dose equivalent (AGDE) in those areas were higher than the limits recommended by ICRP (2021). ^238^U and ^226^Ra have a significant correlation (0.724), and the cluster analysis was showing a statistically meaningful cluster below 5 indicated that they have similar behavior during parent rock weathering and watershed erosion, and the distribution of ^232^Th and ^40^K were influenced by the addition of rock types. The activity ratios of ^226^Ra/^238^U, ^226^Ra/^232^Th, ^238^U/^40^K, and ^226^Ra/^40^K variation indicated that ^40^K more mobile than ^226^Ra and ^238^U, U(VI) was reduced to U(IV) by organic matter in the downstream area and re-entered into the sediment during the sediment surface runoff in the small watershed of the uranium ore open-pit mining area. Therefore, it is necessary to further seal up and repair the tailings landfill area.

## 1. Introduction

All organisms inevitably experience exposure to radioactive radiation throughout their existence on Earth. This radiation primarily originates from radionuclides, and can be classified into artificial radionuclides (such as ^137^Cs, ^239^Pu, and ^134^Cs) and natural radionuclides (such as ^238^U, ^226^Ra, and ^232^Th) [1]. Approximately 85% of the radioactive radiation arises from natural radionuclides [2], which pose a threat to the survival of living organisms. These radionuclides primarily result from natural radioactive decay series (^238^U, ^232^Th, and ^40^K), with a half-life equivalent to the age of the Earth [3]. They are widely distributed in various terrestrial environments, such as soil, sediment, rock, beach, air, vegetation, rivers, and oceans [1,4], and are affected by diverse geological and geographical factors [5].

The worldwide per capita annual effective dose of natural radiation is reported to be 2.4 mSv [1], while in China, it has increased from 2.3 mSv in the early 1990s to around 3.8 mSv by 2016 [6]. The rise in radiation dose resulting from human activities has implications for public health concerns [7]. Anthropogenic activities can increase the concentration of heavy metals and radionuclides [8,9,10]. Activities associated with ore mining and processing can contribute to elevated levels of naturally occurring radioactive materials and heavy metals in soils and sediments [9,10,11,12,13]. Specifically, uranium mining activities involve minerals from uranium deposits that are rich in ^238^U and ^232^Th decay series nuclides, as well as ^40^K [14,15]. During uranium mining and processing, these natural radionuclides can be released from ore minerals [16], leading to media contamination, as well as food and water contamination due to various pollutants originating from mining operations [17,18]. Currently, the global accumulation of uranium tailings exceeds 2 billion tons [14], with only a small fraction of old abandoned tailings ponds having been remediated [6]. According to estimates by the United Nations Scientific Committee on the Effects of Atomic Radiation (UNSCEAR), the collective annual dose to surrounding residents from uranium mines, mills, and tailings is approximately 50–60 person-Sv [14]. Comprehending the migration of radionuclides in uranium ore fields is of paramount importance for safeguarding the local ecological environment and the well-being of residents.

The present study area was one of the old typical granite-type uranium mines in China. The mining and processing of uranium ore have significantly impacted both the normal production activities and the living environment of the local population, leading to increasingly prominent environmental issues [19]. The prolonged mining activities have caused substantial damage to the surface environment within the uranium ore field, exacerbating the environmental challenges faced in the area. Previous studies investigated the activities of ^238^U, ^226^Ra, ^232^Th, and ^40^K in the soil of mining areas and found that the external exposure level of gamma radiation in the uranium mining areas is much higher than the normal value [19,20]. The enrichment mechanism of uranium in the sediment cores of downstream reservoir, adjacent to the uranium deposit, has been investigated using activity ratios of ^230^Th/^234^U and ^230^Th/^238^U, as well as δ^238^U isotopes [21]. However, due to the different geochemical behaviors of ^238^U, ^226^Ra, ^232^Th, and ^40^K during watershed erosion and transport, the enrichment patterns of these nuclides in the area differ. Unfortunately, there has been a lack of research on the redistribution of radioactive nuclides in the soil and sediment surrounding this specific uranium mining region. The present study aims to assess the spatial distribution characteristics of natural radionuclides in the surface soils and river sediments of the uranium mining area, as well as investigate the geochemical features of these radionuclides in the soil and sediments to understand their migration processes. Additionally, the study aims to evaluate the environmental risks associated with radioactivity and provide the geochemical behavior of ^238^U, ^226^Ra, ^232^Th, and ^40^K during the surface runoff in uranium mining areas, and will contribute to the formulation of effective strategies and policies aimed at reducing radiological exposure and ensuring the protection of human health and the environment.

## 2. Methods and Materials

### 2.1. Study Area

**Figure 1:**
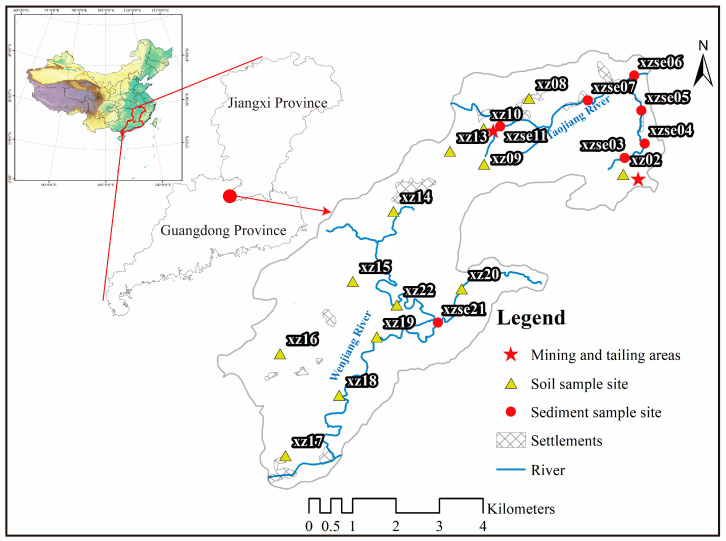
Distribution of sampling points in the study area. The xz represents the topsoil samples and xzse represents river sediment samples.

The uranium deposit area is situated in the neighboring regions of Jiangxi Province and Guangdong Province, characterized by a hilly and mountainous landform and a humid subtropical monsoon climate. The annual average temperature is 22–25 °C. The area experiences significant surface runoff, and Taojiang River runs through it (Figure 1). The local economy primarily relies on economic crops such as rice, forest trees, fruits, metal minerals, small hydropower stations, and animal husbandry. The area under study represents a typical granite-type uranium deposit, and is the largest of its kind in Guangdong Province [22]. It is located in the Guidong granite massif, in northern Guangdong Province, China. The U deposit predominantly consists of medium-grained biotite granite, with Early Jurassic porphyritic and Late Jurassic muscovite microgranite [23]. The ore minerals are predominantly composed of pitchblende, pyrite, hydrogoethite, hematite, and sphalerite, with an average U content of 5–13 mg/kg [24]. The original in situ resources of the deposit were estimated to be between 1500 and 5000 t U, with grades ranging from 0.1–0.3% U [25]. Uranium hydrometallurgical industry was established in 1957, utilizing heap leaching techniques with sulfuric acid [23]. Solid and liquid waste generated from mining and hydrometallurgical activities contain elevated levels of radionuclides as well as various harmful non-radioactive elements, raising concerns about environmental pollution [21]. A mining area restoration project has been initiated since 2019.

### 2.2. Sample Collection and Analysis

In October 2020, samples were collected from the uranium mining area (Figure 1). A total of 21 samples were collected, comprising 8 sediment samples obtained from the opencast mining catchment and 13 surface soil samples. The surface soil samples were collected with dimensions of 10 cm length × 10 cm width × 5 cm depth, and each sample weighed approximately 100 g. River sediments were collected using a box sampler, with a sampling depth of 5 cm and a sample size ranging from 500–1000 g. All samples were carefully placed in resealable plastic bags and transported to the laboratory for further analysis. The soil samples were dried at 60 °C until a constant weight was achieved, while the sediment samples were subjected to freeze-drying until a constant weight was obtained. Each dried sample was finely pulverized by ceramic mortar and passed through a 20 mesh screen for sieving. Subsequently, the samples were securely sealed in plastic centrifuge tubes (60 mm height × 15 mm diameter) [26]. The sealed samples were stored for at least 6 months in a cool, dry spot away from sunlight place for counting to establish a secular equilibrium between ^234^Th and ^238^U to radionuclide analysis [27].

The specific activities of ^238^U, ^226^Ra, ^232^Th, and ^40^K were measured using gamma-ray spectrometry with a highly pure germanium (HPGe) coaxial detector (Canberra GCW3523, CANBERRA Industries Inc, Meriden, CT, USA). To minimize background noise, the detector was shielded by an ultralow background lead cylinder, resulting in a background count of 0.9 counts per second (cps) within the energy range of 10–2000 keV. The detector had an energy resolution of 2.2 keV and a relative detection efficiency of 50.2% [26]. The activity of ^238^U was determined using the gamma line at 63.3 keV for ^234^Th. The activity of ^226^Ra was determined by those of its decay products: ^214^Bi (609 keV) and ^214^Pb (352 keV). The activity of ^232^Th was determined by the ^232^Th decay series of ^228^Ac (338 keV and 911 keV), ^208^Tl (583.2 keV), and ^212^Pb (238.6 keV) [27,28,29]. The activity of ^40^K was directly measured based on the gamma-ray energy peak at 1460 keV. The efficiency calibration of the detector systems was conducted using standard samples (IAEA375 and IAEA-447)(International Atomic Energy Agency, Vienna, Austria) as part of the quality assurance/quality control (QA/QC) method to ensure the reliability of the data. In order to make the uncertainty of each radionuclide less than 10% at the 95% confidence level, the sample counting times were 12–48 h. A procedure blank sample was also measured for 72 h. The radionuclide measurements were analyzed at the Key Laboratory of the Ministry of Education, Qinghai Normal University, Xining, China. The spatial distribution of ^238^U, ^226^Ra, ^232^Th, and ^40^K has been drawn using ArcGIS 10.2(Environmental Systems Research Institute, RedLands, CA, USA).

### 2.3. Calculation of Radiological Indices

In this study, several parameters were calculated to assess the potential risks posed by the radionuclides under investigation in terms of their radiation levels, potential health effects, and the associated carcinogenic risk. These parameters include radium equivalent activity (Ra_eq_), outdoor gamma absorbed dose rate (DR), annual effective dose equivalent (AEDE), annual gonadal dose equivalent (AGDE), and excess lifetime cancer risk (ELCR) were calculated for these radionuclides.

To assess the gamma radiation hazard associated with soil, the radium equivalent activity was used as the radiation hazard index. It is generally accepted that Ra_eq_ should not exceed 370 Bq/kg, and these limits are stated to be equivalent to an effective dose of 1.5 mGy/a based on gamma external radiation dose [1]. The specific formula is as follows:

(1) $${Ra}_{eq}=C_{Ra}+1.43C_{Th}+{0.077C}_{k}$$

The C_Ra_, C_Th_, and C_K_ are the active concentrations (Bq/kg) of ^226^Ra, ^232^Th, and ^40^K, respectively.

Based on the activity levels of ^226^Ra, ^232^Th, and ^40^K, the absorbed gamma dose rate (DR, nGy/h) of terrestrial gamma radiation in the air at 1 m level was calculated. It is calculated as follows [1]:

(2) $$DR=0.462C_{Ra}+0.604C_{Th}+0.0417C_{K}$$

The C_Ra_, C_Th_, and C_K_ are the active concentrations (Bq/kg) of ^226^Ra, ^232^Th, and ^40^K, respectively [1].

The annual effective dose equivalent (AEDE, mSv/y) is an assessment of potential biological effects associated with populations exposed to ionizing radiation, and effective dose rates are used for radiation protection. This study calculates the outdoor annual effective dose equivalent. Its calculation formula is as follows [1]:

(3) AEDE=DR×DCF×OF×T

The DR is the absorbed gamma dose rate, DCF is the dose conversion coefficient (0.7 Sv/Gy), OF is the outdoor occupancy coefficient (20%), and T is the time coefficient (8760 h) [1].

Annual gonadal dose equivalent (AGDE, μSv/y) is a measure of the genetic significance of the annual dose equivalent received by the reproductive organs (gonads) of the population. The relationship is as follows [30]:

(4) $$AGDE=3.09C_{Ra}+4.18C_{Th}+0.314C_{K}$$

The C_Ra_, C_Th_, and C_K_ are the active concentrations (Bq/kg) of ^226^Ra, ^232^Th, and ^40^K, respectively [30].

The lifetime risk of fatal cancer is related to the likelihood of developing lifetime cancer at a certain exposure [28]. Excess lifetime cancer risk (ELCR) estimates the probability that a human population will develop cancer from exposure to naturally occurring radionuclides during a given lifetime. This is calculated using the following formula [31]:

(5) ELCR=AEDE×DL×RF

where DL is the life expectancy (70 years), and RF is the fatal risk factor per year, equal to 0.057 Sv^−1^ [1,31].

The spatial distribution of Ra_eq_, DR, AEDE, AGDE, and ELCR has been drawn using ArcGIS 10.2.

## 3. Results and Discussion

### 3.1. Radioactivity of Radionuclides

**Figure 2:**
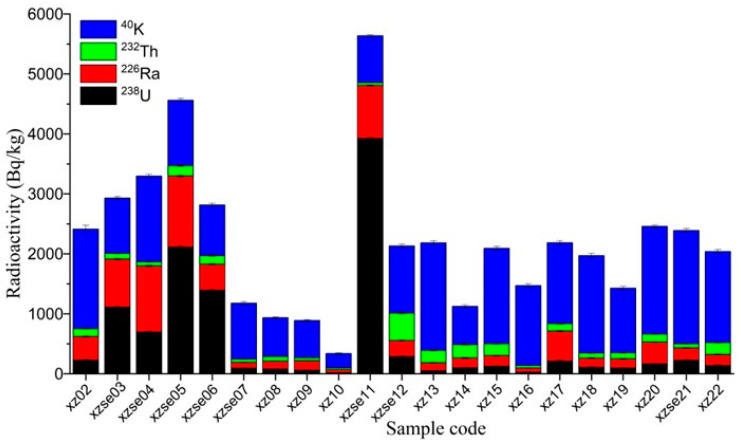
The variation in ^238^U, ^226^Ra, ^232^Th, and ^40^K in the sample of study areas.

The activities of natural radionuclides (^238^U, ^226^Ra, ^232^Th, and ^40^K) in the surface soil and sediments of the study area are depicted in Figure 2. Among them, the highest value of ^238^U is the tailings pond landfill area (xzse11) at 3925 ± 20 Bq/kg, while the lowest value of 17 ± 2 Bq/kg is observed in the soil upstream of the tailings pond (xz10). The activity of ^226^Ra is 50–1180 Bq/kg, with the highest value recorded in xzse05 and the lowest value in xz10. The activity of ^232^Th ranges from 29–459 Bq/kg, and that of ^40^K ranges from 240–1890 Bq/kg. The average activities of ^238^U, ^232^Th, ^226^Ra and ^40^K were 537 ± 966 Bq/kg, 130 ± 98 Bq/kg, 360 ± 351 Bq/kg, and 983 ± 382 Bq/kg, respectively. These average values not only exceed the soil measurements reported by a previous study [20] in the same area, but are also significantly higher than the global average values (35 Bq/kg for ^238^U, 35 Bq/kg for ^226^Ra, 30 Bq/kg for ^232^Th, and 400 Bq/kg for ^40^K [1], and the average values for China (33 Bq/kg for ^238^U, 32 Bq/kg for ^226^Ra, 41 Bq/kg for ^232^Th, and 440 Bq/kg for ^40^K [1]. Furthermore, these values also exceed the average values of these radionuclides in the Earth’s upper crust (33 Bq/kg for ^238^U, 33 Bq/kg for ^226^Ra, 43 Bq/kg for ^232^Th, and 720 Bq/kg for ^40^K [32]. The variation coefficient, which indicates the degree of variation or heterogeneity, follows the order: ^238^U (180%) > ^226^Ra (97%) > ^232^Th (76%) > ^40^K (39%). This implies that the distribution of ^238^U and ^226^Ra in the study area is highly heterogeneous, likely influenced by the distribution of uranium deposits and human activities such as mining [33]. On the other hand, the variation coefficient for ^40^K is relatively small, indicating a more stable and less affected distribution on the surface, less influenced by lithology and human activities (mining activities).

### 3.2. Spatial Distribution Characteristics of Radionuclides

**Figure 3:**
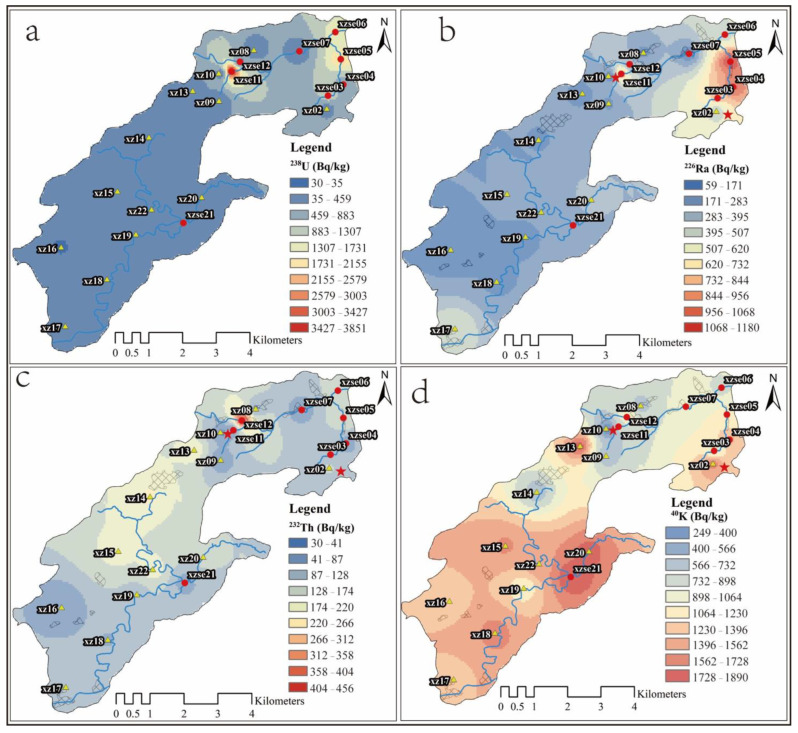
Spatial distribution characteristics of ^238^U (**a**), ^226^Ra (**b**), ^232^Th (**c**), and ^40^K (**d**) using inverse distance weighted spatial analysis. The red five-pointed stars represent mining areas and tailings ponds, the red circles represent sediment samples, and the yellow triangles represent topsoil samples.

The spatial distribution of radionuclides, obtained through inverse distance weighted spatial analysis, is presented in Figure 3. The activity of ^238^U exhibits an increasing trend from the southwest to the northeast in the study area. High activity levels are observed in the open-pit mining area (xz02–xe06) and the tailings area (xzse11). Samples from the small watersheds, xzse03 to xzse06, generally show high ^238^U activity (Figure 3a). This suggests that the open-pit uranium mining area plays a significant role in the distribution of ^238^U, due to the production of a large amount of slag during the mining process, which then spreads to the surrounding soil. The surface vegetation in the mining area has been extensively damaged, leading to increased soil erosion in the watershed. As a result, a substantial amount of highly concentrated uranium soil has rapidly migrated downstream through surface runoff, intensifying soil erosion. This, in turn, contributes to the high activity of U in the river sediment within this catchment. Although pit closure and mine restoration measures have been implemented, the restoration efforts have mainly focused on the xz02 to xzse03 area during the sampling period. Corresponding treatment has not been carried out in the middle and lower reaches of the watershed. Consequently, the upstream area of the small watershed exhibits lower ^238^U values compared to the downstream area. This is because the soil used for mine restoration is low-background soil containing uranium, which is transported from other locations. During surface runoff, a portion of this soil may erode and be transported downstream, diluting the original high-uranium sediment. As a result, the ^238^U activity in the sediment tends to increase from upstream to downstream. Since ^226^Ra is a decay product of ^238^U, its spatial distribution characteristics are similar to those of ^238^U, showing an increasing trend from the southwest to the northeast in the study area, with high values observed in the uranium open-pit mining area. The changes in ^226^Ra closely resemble those of ^238^U in the small watersheds of the open-pit mining area in the northeast. This indicates that the mine restoration efforts by the engineering team have exerted a certain control over the pollution of these two radionuclides.

The high activity of ^232^Th is observed in the soil samples located in the southwest of the study area, specifically in xz13–xz15 and xz22 (Figure 3c). The trend of ^232^Th activity shows a decreasing pattern from the south-central to the northeast in the study area. On the other hand, the high activity of ^40^K is predominantly distributed in the southern part of the study area (Figure 3d). As one moves from the south to the north, the ^40^K activity generally exhibits a decreasing trend. Interestingly, in the small watershed of the uranium mining area within the open-pit mining zone, the distribution characteristics of ^40^K are completely opposite to those of ^238^U, ^226^Ra, and ^232^Th. The activity of ^40^K tends to decrease from upstream to downstream in this particular area.

The high values of ^238^U and ^226^Ra in the study area can be attributed to the influence of uranium mining and tailings. Additionally, the spatial distribution of radionuclides is also controlled by the lithology [34] and geochemistry condition [35]. In the east of the Guidong granite body, where the uranium deposit is located, the Maofeng, Sundong, and Xiazhuang granites serve as the main host rocks for the ore field [36]. The surrounding rocks in the northeastern part of the Guidong rock body consist of Cambrian–Ordovician shallow metamorphic sandstone, shale, and slate [24], while the southwestern part is primarily composed of Devonian–Carboniferous siltstone, sandstone, glutenite, and carbonate rock [19]. The source rocks of the Guidong pluton contain ancient uranium-rich layers [37], and the Sinian–Cambrian rock layers have high uranium and thorium contents, reaching 36 mg/kg and 19.5 mg/kg, respectively [38]. The elevated uranium and thorium content in the rock formations contributes to the high radioactivity of ^238^U, ^226^Ra, and ^232^Th in the study area. Potassium (K) is mainly enriched in the alkaline feldspar and mica during the late stage of magma evolution in this area. As the magma evolves, the basic components (Ca, Mg, and Fe) gradually decrease, while the acid–base components (Si, K, Na, and Li) increase [37]. The lithology of the rock mass transitions from acidic to intermediate acidic to super acidic in the early stage, with subsequent intrusion of intermediate-basic magma and intermediate-acid volcanic activity in the late stage. In terms of uranium content, the rocks exhibit an increasing trend from the early period to the later period [24]. As a result, the activity of ^40^K tends to be higher in the southern part of the study area.

### 3.3. Radiological Hazards

**Figure 4:**
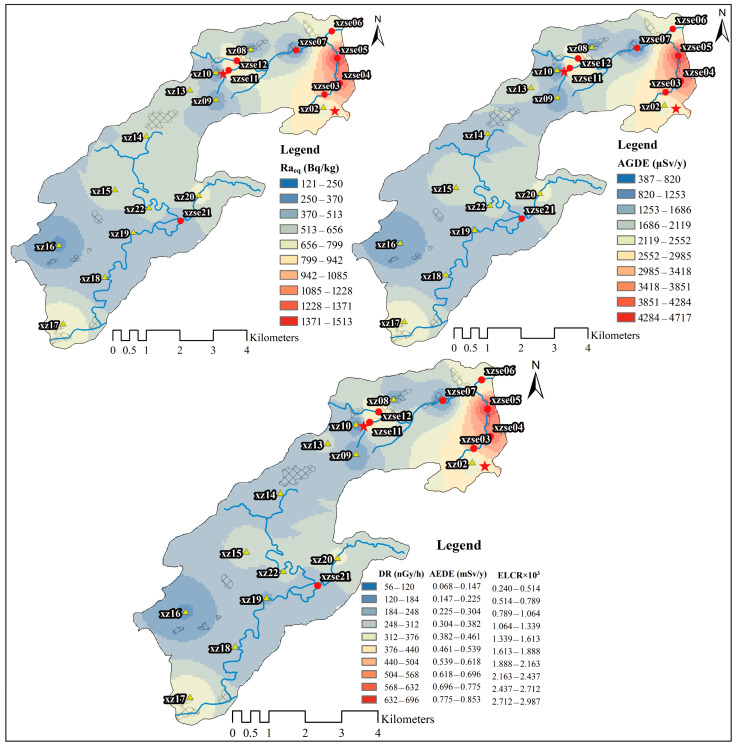
Spatial distribution characteristics of radiological hazards using inverse distance weighted spatial analysis. The red five-pointed stars represent mining areas and tailings ponds, the red circles represent sediment samples, and the yellow triangles represent topsoil samples.

The spatial distribution of Ra_eq_ in the study area is shown in Figure 4, with values ranging from 112–1513 Bq/kg. The high-value areas are primarily located in the open-pit mining and tailings areas. The Ra_eq_ values in these areas are more than two times higher than the reference value of 370 Bq/kg recommended by UNSCEAR [1]. There are five sampling sites (23% of the total) with Ra_eq_ values below 370 Bq/kg, mainly concentrated in the central part of the research area. The presence of high R_aeq_ values indicates that the study area has a high natural gamma radioactivity geological background. The relative contributions of ^226^Ra, ^232^Th, and ^40^K to Ra_eq_ vary significantly across different regions. In the open-pit mining catchment (xzse02 to xzse06), the relative contribution of ^226^Ra to Ra_eq_ ranges from 26–87%, with an average value of 60%. There is a strong relationship between Ra_eq_ and ^226^Ra in the open-pit mining catchment, with a correlation coefficient of 0.919 (*p* < 0.01). This suggests that the variation in ^226^Ra primarily controls the characteristics of Ra_eq_ in the open-pit mining catchment. In other sampling sites, the relative contributions of ^226^Ra and ^232^Th to Ra_eq_ range from 23–64% and 22–61%, respectively, both with average values of 38%. However, there is a significant correlation between ^226^Ra and Ra_eq_ (correlation coefficient of 0.823; *p* < 0.01). Therefore, in the study area, the variation in ^226^Ra has a dominant influence on the variation in Ra_eq_.

The spatial distribution of the AGDE is shown in Figure 4. The values of AGDE range from 355–4718 μSv/y, which is higher than the worldwide average (300 μSv/y) [1]. In the open-pit mining area, the AGDE varies from 2287–4718 μSv/y, which is more than 7.5 times higher than the world average. The relative contribution of ^226^Ra to the AGDE values ranges from 22–86%, with an average contribution of 48%. There is a significant correlation between ^226^Ra and AGDE (Pearson correlation coefficient of 0.917; *p* < 0.01), indicating that the variation in ^226^Ra controls the spatial distribution of AGDE in the study areas.

The ranges of DR, AEDE, and ELCR in the study areas ranged from 51–696 nGy/h, 0.063–0.854 mSv/y, and 0.220 × 10^−3^–0.299 × 10^−2^, respectively (Figure 4). With the exception of sampling site xz10, the DR, AEDE and ELCR values are much higher than the average world values reported by UNSCEAR [1] (59 nGy/h, 0.070 mSv/y and 0.290 × 10^−3^, respectively). The high values of DR, AEDE, and ELCR are predominantly distributed in the open-pit mining and tailings areas. In these areas, the DR, AEDE, and ELCR values are approximately 6 times, 5.5 times, and 5 times higher than the average world values reported by UNSCEAR [1], respectively. Overall, the relative contribution of ^226^Ra to DR, AEDE, and ELCR values ranges from 23–83%, with an average value of 50%. There is a significant correlation between ^226^Ra and DR, AEDE, and ELCR values (correlation coefficient of 0.929; *p* < 0.01), indicating that ^226^Ra predominantly influences the spatial distribution of DR, AEDE, and ELCR in the study areas.

Given the high radioactivity levels of ^226^Ra, ^232^Th, and ^40^K in the open-pit mining areas and tailings areas, the values of the AGDE exceed the limits recommended by ICRP [39] and the general limit of 1 mSv/y. This suggests that protective measures should be implemented to minimize radiological exposure for individuals living in those areas and during mining activities. To address the impact of radionuclides in the uranium mining area on the surrounding environment, environmental remediation measures are necessary. This includes not only soil restoration in the mining area, but also the cleanup and containment of river sediments in the affected areas. The high values of radioactive indicators in the downstream areas of the tailings area may be attributed to flash floods and leaks from the sealed tailings, which can enter rivers through surface runoff and consequently affect the aquatic environment. Therefore, it is crucial to implement measures to further seal and rehabilitate the tailings landfill area to prevent further contamination and minimize environmental risks.

Comparisons of the mean values of Ra_eq_, DR, AEDE, AGDE, and ELCR in the mining areas of the present study with similar research conducted worldwide for uranium ore mining, as well as the levels recommended by UNSCEAR [1], are shown in Table 1. 

The highest values of Ra_eq_, DR, AEDE, AGDE, and ELCR were found in Laocai, Vietnam. This can be attributed to the presence of placer ores containing minerals such as thorium, monazite, oxinite, checchite, bastnezite, smacskite, manhetite, ilmenite, zircon, and barite, which have high concentrations of Th and U (with mean mass fractions of 0.157% and 0.016%, respectively) [47]. The lowest radiological hazard values were found in Manyoni, Tanzania [43], where the host rocks are dominated by gneiss–granite–migmatite complex geology [43], which may have concentrations of Ra, Th, and K. The values of radiological hazards in the Xiazhuang uranium deposit fall within the range of previous studies.

### 3.4. Multivariate Statistical Analysis

The values of radionuclides and radiological indices has been normalized before conducting the multivariate statistical analysis. The Pearson correlation coefficient analysis in Table 2 indicates the relationships between radionuclides and radiological hazard parameters. It shows that ^238^U and ^226^Ra have a significant correlation (0.724; *p* < 0.01). As ^226^Ra is the decay product of ^238^U [1], this high correlation suggests that ^226^Ra inherits characteristics from ^238^U [26], indicating that both radionuclides have similar behavior during parent rock weathering and watershed erosion [47]. The lower correlations observed among ^40^K, ^232^Th, and ^238^U series suggest that these radionuclides may have different sources [48] and undergo different transport processes during surface runoff. This can be attributed to variations in the host minerals of uranium, thorium, and potassium, which are different in the study area; the high value of ^238^U in the study area being distributed in the mining and tailings pond regions; and the area containing uranium-rich minerals with high concentrations, such as asphaltic [19,37]. ^232^Th, on the other hand, is generally more stable compared to ^238^U, and its content is primarily associated with heavy minerals, such as uranothorite and monazite [37]. The distribution of ^40^K is mainly influenced by potassium-rich minerals, such as potassium feldspar. The rock types in this region transition from medium coarse grained porphyritic biotite granite to fine grained porphyritic biotite granite as one moves from southwest to northeast. The decreasing trend of potassium-rich minerals in the rocks also contributes to a similar trend in ^40^K. Consequently, the low of correlation among ^238^U, ^232^Th, and ^40^K indicates that these elements are controlled by the geological characteristics and minerals of the study areas. The uranium was in the mining areas and tailings pond because of the pitchblende, Th was enriched here, and K was enriched potash feldspar [24,49]. The relationship between these radionuclides indicates that their distribution is influenced by the specific host minerals present in the study area. The significant correlations between the radioactive indicators and ^226^Ra further emphasize that the activity of ^226^Ra controls the variations observed in the radiological hazard parameters. This suggests that monitoring and assessing the activity of ^226^Ra can serve as a reliable indicator for evaluating radiological hazards in the study area.

**Figure 5:**
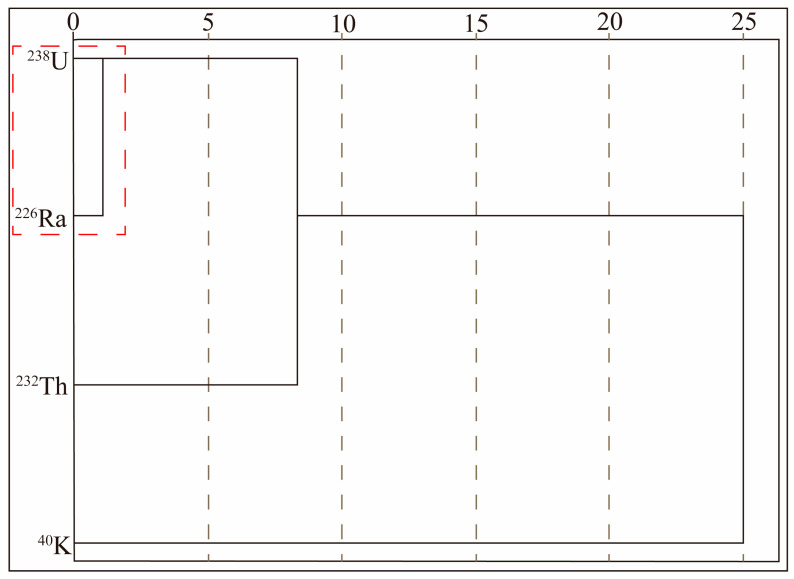
Cluster analysis of radionuclides. The red box was the significant concentration.

The cluster analysis based on the Ward method and square Euclidean distance for radioactive variables in the uranium deposit (Figure 5) revealed three distinct clusters. The first cluster consisted of ^238^U and ^226^Ra, showing a statistically significant concentration below a square Euclidean distance of 5. The second cluster comprised ^232^Th, and the third cluster represented ^40^K. These cluster results align with the Pearson correlation analysis, further confirming the associations among the radionuclides. The findings of the cluster analysis and correlation relationships highlight that the spatial distribution heterogeneity of radionuclides is primarily governed by their geochemical processes and the regional geographical environment [38]. This is consistent with the influence of different rock types on the distribution of radionuclides [50]. The geochemical behavior of natural radionuclides varies in response to common geological processes within the study area. This suggests that the interaction between radionuclides and their surrounding environment, including rock types and geochemical conditions, plays a significant role in shaping their spatial distribution patterns.

### 3.5. Radioactivity Ratio of Radionuclide

**Figure 6:**
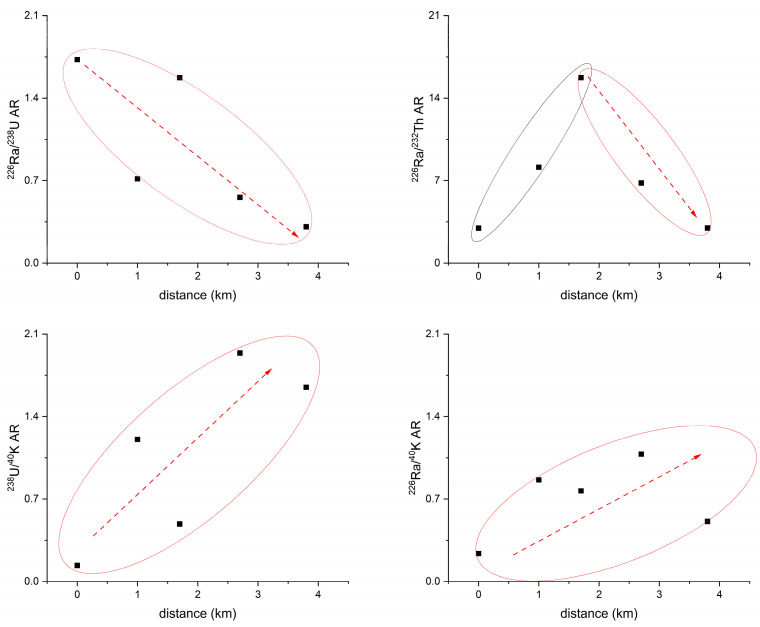
Changes in the ratio of radionuclide activity from upstream to downstream in the open-pit mining area. The black square was the value of radionuclide activity and the dotted line was the trend of those values.

The trends of radioactivity ratios ^226^Ra/^238^U (^226^Ra/^238^U AR), ^226^Ra/^232^Th (^226^Ra/^232^Th AR), and ^238^U/^40^K (^238^U/^40^K AR), and ^226^Ra/^40^K (^226^Ra/^40^K AR) with respect to the distances from sampling sites xz02 to xzse06 in the open-pit mining area are shown in Figure 6. The ^226^Ra/^238^U AR exhibited a general decreasing trend from the mining area towards the downstream in Taojiang River. In the mining area, the ^226^Ra/^238^U AR was above 1.5. The host mineral for uranium in the area was primarily pitchblende [19,37]. During mining activities and soil erosion processes, the valence state of uranium changed from U(IV) to U(VI) under oxidizing conditions, leading to enhanced mobility of uranium [51]. On the other hand, radium has a strong affinity for particles in freshwater environments [52,53,54]. The values of ^226^Ra/^238^U AR in the soil of the mining areas indicated that uranium was more easily removed compared to radium. The conversion of uranium from U(IV) to U(VI) occurred in the mining area due to weathering and mining activities in the upstream source area. As a result, uranium migrated downstream towards the middle and lower reaches. The enrichment of uranium ore appeared to be positively correlated with organic matter [55]. Due to the presence of a large pig farm in the middle reaches of this watershed (the site was between xzse04 and xzse05), the waste discharged from the pig farm contains a significant amount of organic matter (observed on-site), leading to an increase in the downstream area’s organic matter content. The enrichment of organic matter has led to the sediment becoming an anaerobic environment. In this environment, organic matter act as electron acceptors, facilitating the reduction of dissolved U(VI) to U(IV), and the organic matter provides adsorption sites for U(IV) [56], transforming it from a dissolved state to a particulate state and re-entered into the sediment, leading to a decrease in the ^226^Ra/^238^U AR from the source area to the downstream. Overall, the observed trends in the ^226^Ra/^238^U AR indicate the influence of weathering, mining activities, and the presence of organic matter on the mobility and distribution of uranium and radium in the study area.

The ^226^Ra/^232^Th AR ratio exhibited an initial increase followed by a decrease (Figure 6). However, it showed a decreasing trend in sediments. This behavior can be explained by the differential migration abilities of their parent radionuclides, ^238^U and ^232^Th, during surface runoff processes in Taojiang River [26]. ^238^U has greater mobility than ^232^Th and can migrate in dissolved form. On the other hand, ^232^Th is relatively stable and can only migrate under strong acid conditions [50]. As a result, the ^226^Ra/^232^Th AR ratio showed a decreasing trend in the midstream and downstream during surface runoff processes. ^238^U/^40^K AR and ^226^Ra/^40^K AR showed an increased trend during surface runoff in the open-pit mining areas. During weathering, potassium bearing minerals can readily release K^+^ ions, which can be easily transferred because the solubility of potassium gives it high mobility during surface geological processes [50]. The high mobility of ^40^K in the supergene process [48] can easily become activated and migrate with surface runoff during mining processes [26]. Consequently, in the small watershed of the open-pit mining area, the ^40^K concentration showed a decreasing trend from upstream to downstream due to its high migration and emigration [57]. Therefore, the mining process can accelerate the migration rate of radionuclides, such as ^238^U, ^226^Ra, and ^232^Th, from upstream to downstream. However, the subsequent remediation efforts in uranium mines have slowed down the release rate and flux of these radionuclides downstream, leading to the observed trends in their spatial distribution.

## 4. Conclusions

The present study described the concentrations, spatial distributions, and assessments of radiological hazards for natural radionuclides in the sediments and surface soil of a type of granite uranium mining area in China. The distributions of ^238^U and ^226^Ra are controlled by uranium mining areas, while the spatial distribution of ^40^K is controlled by magma evolution. The average activities of ^226^Ra, ^238^U, ^232^Th, and ^40^K were much higher than the world safe value. High values of Ra_eq_, DR, AEDE, AGDE, and ELCR are all located in the studied uranium mining and tailings areas, among them, due to the impact of uranium mining, these indicators in uranium mining areas are more than twice the world average, and even the highest can reach more than 10 times. Therefore, workers or local residents operating in this area need to take corresponding protective measures. ^238^U and ^226^Ra have a significant correlation (0.724), and the cluster analysis was showing a statistically meaningful cluster below 5 indicated that they have similar behavior during parent rock weathering and watershed erosion and the distribution of ^232^Th and ^40^K were influenced by the addition of rock types. The activity ratios of ^226^Ra/^238^U, ^226^Ra/^232^Th, and ^238^U/^40^K variation indicated that ^40^K was more mobile than ^226^Ra and ^238^U in the small watershed of the uranium ore open-pit mining area. Due to the rich content of organic matter in the downstream area, U(VI) was reduced to U(IV) by organic matter in the downstream area and re-entered into the sediment. This study aimed to provide fundamental information regarding the concentrations, distributions, and potential hazards of radionuclides in uranium mining area in China. It can contribute to the formulation of policies aimed at minimizing the adverse effects on human health and the environment and potential impact and ensure the protection of both human populations and the surrounding ecosystem.

## Figures and Tables

**Table 1:** Radiological hazards on uranium deposit in previous studies from other regions in comparison to this study.

Ra_eq_ (Bq/kg)	DR (nGy/h)	AEDE (mSv/y)	AGDE (μSv/y)	ELCR	Country	Reference
24,802	11,447	14	76,608	49.1 × 10^−3^	Australian	[40]
781	362	0.444	2458	1.55 × 10^−3^	Kangra, India	[41]
617	289	0.355	1979	1.24 × 10^−3^	Salamanca, Spain	[42]
329	149	0.183	1009	0.639 × 10^−3^	Manyoni, Tanzania	[43]
466	213	0.261	1470	0.913 × 10^−3^	Egypt	[44]
3309	1531	1.88	10,274	6.57 × 10^−3^	Köprübaşi, Türkiye	[45]
12,078	5545	6.8	37,184	23.8 × 10^−3^	Jiangxi, China	[46]
27,508	11,736	14.4	80,976	50.4 × 10^−3^	Laocai, Vietnam	[47]
1049	485	0.594	3301	2.08 × 10^−3^	Xiazhuang, China	This study
370	59	0.07	300	0.290 × 10^−3^	World average	[1]

**Table 2:** Pearson correlation of each nuclide and radionuclide index.

	^238^U	^226^Ra	^232^Th	^40^K	Ra_eq_	DR	AEDE	AGDE	ELCR
^238^U	1	0.724 **	−0.112	−0.229	0.610 **	0.615 **	0.615 **	0.606 **	0.615 **
^226^Ra		1	−0.046	0.003	0.915 **	0.924 **	0.924 **	0.917 **	0.924 **
^232^Th			1	0.181	0.350	0.323	0.323	0.335	0.323
^40^K				1	0.168	0.180	0.180	0.195	0.180
Ra_eq_					1	0.999 **	0.999 **	0.999 **	0.999 **
DR						1	1.000 **	1.000 **	1.000 **
AEDE							1	1.000 **	1.000 **
AGDE								1	1.000 **
ELCR									1

** *p* < 0.01.

## Data Availability

Data are contained within the article.
